# One century later: the folk botanical knowledge of the last remaining Albanians of the upper Reka Valley, Mount Korab, Western Macedonia

**DOI:** 10.1186/1746-4269-9-22

**Published:** 2013-04-11

**Authors:** Andrea Pieroni, Besnik Rexhepi, Anely Nedelcheva, Avni Hajdari, Behxhet Mustafa, Valeria Kolosova, Kevin Cianfaglione, Cassandra L Quave

**Affiliations:** 1 University of Gastronomic Sciences, Piazza Vittorio Emanuele 9, Pollenzo/Bra, (Cuneo), I-12042, Italy; 2 Department of Biology, State University of Tetova, Ilindenska, Tetovë, Republic of Macedonia; 3 Department of Botany, University of Sofia, Blv. Dragan Tzankov 8, Sofia, 1164, Bulgaria; 4 Department of Biology, University of Prishtina “Hasan Prishtina”, Mother Teresa Str, Prishtinë, 10 000, Republic of Kosovo; 5 Institute for Linguistic Studies, Russian Academy of Sciences, Tuchkov pereulok 9, Saint Petersburg, 199053, Russia; 6 School of Environmental Sciences, University of Camerino, Via Pontoni 5, Camerino (Macerata), I-62032, Italy; 7 Center for the Study of Human Health, Emory University, 550 Asbury Circle, Candler Library 107E, Atlanta, GA, 30322, USA

**Keywords:** Ethnobotany, Mavrovo, Traditional Knowledge, Balkans

## Abstract

**Background:**

Ethnobotanical surveys of the Western Balkans are important for the cross-cultural study of local plant knowledge and also for obtaining baseline data, which is crucial for fostering future rural development and eco-tourism initiatives in the region. The current ethnobotanical field study was conducted among the last remaining Albanians inhabiting the upper Reka Valley at the base of Mount Korab in the Mavrovo National Park of the Republic of Macedonia.

The aims of the study were threefold: 1) to document local knowledge pertaining to plants; 2) to compare these findings with those of an ethnographic account written one century ago and focused on the same territory; and 3) to compare these findings with those of similar field studies previously conducted in other areas of the Balkans.

**Methods:**

Field research was conducted with all inhabitants of the last four inhabited villages of the upper Reka Valley (n=17). Semi-structured and open interviews were conducted regarding the perception and use of the local flora and cultivated plants.

**Results and conclusion:**

The uses of ninety-two plant and fungal taxa were recorded; among the most uncommon uses, the contemporary use of young cooked potato (*Solanum tuberosum*) leaves and *Rumex patientia* as a filling for savory pies was documented. Comparison of the data with an ethnographic study conducted one century ago in the same area shows a remarkable resilience of original local plant knowledge, with the only exception of rye, which has today disappeared from the local foodscape. Medicinal plant use reports show important similarities with the ethnobotanical data collected in other Albanian areas, which are largely influenced by South-Slavic cultures.

## Background

Ethnobiological studies conducted in the Western Balkans in recent years have reported a rich biocultural diversity and a remarkable vitality of traditional knowledge (TK) concerning the local flora in this region [1-12]. Such studies have been postulated to represent crucial lynch-pins for the development of community-based management strategies for local natural resources, sustainable eco-tourism and high-quality niche food and herbal products [13].

On the other hand, the ethno-historical perspective in the European ethnobotanical literature may represent an important tool for exploring trajectories of changes in plant use, as a few recent works have shown [14-18]. However, the integration of original ethnographic data with historical reports can only take place in those areas in Europe where detailed reports on *local* uses of plants are available. The comparison of current ethnographic data on plant uses with that reported in ancient treatises on medicinal plants can be more complex and even problematic, as information regarding *local* plant perceptions cannot generally be traced back. Comparative analysis between the plant knowledge of historical medical schools and that of subaltern rural classes may, however, be useful for understanding eventual hybridisations of these diverse plant knowledge systems [19-21].

The upper Reka Valley in Western Macedonia represents one of the very few Albanian-speaking areas in South Eastern Europe where a very detailed ethnographic account – including important notes concerning local food and medicinal plant uses - was written in the first decade of the 20th Century. Bajazid Elmaz Doda (approx. 1888–1933) was the personal assistant and long-term partner of one of the most famous scholars in the field of Albanian studies: the Hungarian aristocrat and palaeontologist Baron Franz Nopcsa von Felső-Szilvás (1877–1933). Doda finalised a manuscript in 1914, probably written in collaboration with his mentor/partner, which was focused on the daily mountain life of his village, Shtirovica, located in the upper Reka Valley (approx. 1400 m.a.s.l.). This manuscript remained unpublished until the Albanologist Robert Elsie found it in the Austrian National Library and edited it in 2007 [22]. Doda apparently wrote this account to challenge the argument of the Serbian-Austrian historian and astronomer Spiridon Gopčević (1855–1928), who described the Albanians of the upper Reka Valley as “albanicised Slavs” [23].

Doda’s village of Shtirovica was completely destroyed in 1916 by the Bulgarian army [22]. However, a few surrounding tiny Albanian villages still survive to this day, despite the fact that the local population has been dramatically eroded by recent migration waves, both to the main centres in Macedonia and also abroad.

The aim of this study was to record the traditional plant knowledge of the last remaining Albanians living in these villages of the upper Reka Valley and to compare this with the ethnobotanical notes found in Doda’s work in order to better understand trajectories of change in plant uses. Moreover, a further objective of the study was to compare this field data with that of other recent ethnobotanical surveys conducted in surrounding areas and countries in order to trace commonalities and similarities, and to address overlaps and divergences in Albanian and South-Slavic traditional plant knowledge and practice.

## Methods

### Field study

**Figure 1:**
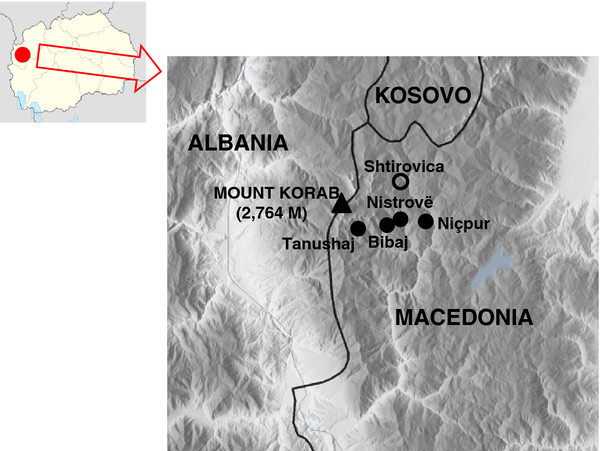
Study area.

In-depth open and semi-structured interviews, as well as participant observation were conducted in August 2012 with members (n=17) of all remaining families of the last inhabited villages of the upper Reka Valley (Figure 1): Nistrovë, Bibaj, Niçpur, and Tanushaj, within the Mavrovo National Park. The same villages were inhabited a few decades ago by hundreds of locals, who mostly migrated to the nearby towns of Gostivar and Skopje, as well as abroad for work or (as in Tanushaj) as a consequence of a (minor) Macedonian portion of the last Yugoslavian Wars.

Locals are now exclusively Muslims, but Albanians of Christian Orthodox faith also lived in the villages until a few decades ago. For example, in Nistrovë, one side of the village (with a mosque) is inhabited by Muslims, while the other side was inhabited by Orthodox believers. The entire population of Orthodox Christians migrated to towns a few decades ago, but they return to their village homes sometimes during the summer. Most of the houses in this part of the village are however abandoned even though the Church has been recently restored. According to our (Albanian Muslim) informants, these migrated Orthodox Christian Albanians assimilated within the Macedonian culture and now prefer to be labelled as “Macedonians”, even if they are still able to fluently speak Albanian. Contact between these two subsets of the village communities, which were very intense and continuous in the past, no longer exists today.

All Albanian inhabitants of the upper Reka are – to different degrees depending on the age – bilingual in Macedonian. Participants were questioned about traditional uses of medicinal plants and wild food plants (in use until a few decades ago or still in use today). Specifically, data concerning the local name(s) of each quoted taxon, the plant part(s) used, in-depth details about its/their manipulation/preparation and medicinal or food use(s) were collected. Interviews were conducted in Albanian with the help of two simultaneous translators.

Prior informed consent was always obtained verbally before conducting interviews and researchers adhered to the new ethical guidelines of the American Anthropological Association [24]. During interviews, informants were always asked to show the quoted plants. Voucher specimens of the most uncommon wild taxa, as well as digital pictures of the most quoted preparations were taken and are deposited at the University of Tetovo and at the University of Gastronomic Sciences, respectively. A short video documentation of the field study is available online [25].

Taxonomic identification was conducted by the first author and plant nomenclature follows *Flora Europaea*[26], the Angiosperm Phylogeny Group III system [27] and The Plant List database [28]. The collected data was compared with Bajazid Elmaz Doda’s ethnographic study, which was conducted one century ago in the village of Shtirovica (Figure 1), within the same study area of our survey [22], and with the most relevant recent Balkan ethnobotanical field studies [1,8-10,13,29-33] and the other available South-Slavic linguistic and folkloric-botanical sources [22,34-44].

## Results and discussion

### The current ethnobotanical knowledge of the upper Reka

Table 1 reports the plant uses recorded in the upper Reka Valley. Ninety-two taxa were reported to be known and in use by the last remaining inhabitants, who were all interviewed. The resilience of the local traditional knowledge concerning plants is especially remarkable when compared with the recordings of the local plant knowledge documented one century ago (see last column of the table [22]). A few of the plant uses (with the exception of rye) recorded one century ago are still actively practiced today in the upper Reka Valley.

**Table 1:** Folk names and uses of plants and fungi quoted in the current study, compared with those recorded one century ago in the same area

**Scientific taxon and family**	**Local folk name(s)**	**Ecological status or provenience**	**Part(s) used**	**Local use(s)**	**Folk name(s) and use(s) as recorded one century ago in the same area [**[22]**]**
*Abies alba* Mill. and *Picea abies* (L.) H. Karst. (Pinaceae)	*Bren*	W	Resin (smol*)	MEDICINAL: topically applied to wounds, sometimes together with tobacco (as haemostatic) or on warts	*Breh* MEDICINAL: resin (smol*) as an ingredient of a home-made poultice (*mehlem*) - made also by adding wax, fat, and powdered pine wood – for treating wounds
*Acer pseudoplatanus* L. (Sapindaceae)	Klenje*	W	Wood	HANDICRAFTS: diverse objects, among them, snow shoes	*Pani*
	Kleni*				
			Bark	VETERINARY: decoction, in external washes for treating wounds in animals	
*Achillea millefolium* L. (Asteraceae)	*Lule e bardhë*	W	Dried flowering aerial parts	MEDICINAL: tea, considered healthy for stomach-ache and liver problems; traded in the past	
	*Lule miu*				
*Allium cepa* L. (Amaryllidaceae)	*Qepa*	C	Bulbs	FOOD: many culinary uses, including home-made savory pies called *ndri*, filled with buttermilk (*dhallët*) and diverse vegetables; MEDICINAL: compresses made with crushed onions and salt for treating bruises RITUAL: burned on the fire	*Qep* FOOD: filling for savory pies MEDICINAL: externally applied with salt on wounds
*Allium porrum* L. (Amaryllidaceae)	Prash*	C	Fresh aerial parts	FOOD: filling for home-made savory pies (*ndri*)	*Prasa*
			Juice	MEDICINAL: instilled in the ear for treating ear-ache	
*Allium sativum* L. (Amaryllidaceae)	*Hudra*	C	Bulbs	FOOD: seasoning	*Hudr*
				RITUAL: burned on the fire; the resulting strong odour was considered a repellent for werewolves; tied to cow horns as a protective amulet against evil-eye	
*Alnus glutinosa* (L.) Gaertn. (Betulaceae)	*Verri*	W	Bark	DYEING: the bark was boiled in the past; the resulting red decoction was used for dyeing in black	*Verri*
*Amaranthus* spp. (Amaranthaceae)	*Llabot e egër*	W	Leaves	FODDER	
*Arctium lappa* L. (Asteraceae)	*Kakuda*	W	Leaves	FODDER	
*Atriplex hortensis* L*.* (Amaranthaceae)	Laboda*	C	Leaves	FOOD: most preferred filling for pies (*ndri*)	
	Labat*				
*Betula pendula* Roth (Betulaceae)	*Mustekna*	W	Bark	MEDICINAL: burned; the vapours are exposed to the skin for treating skin inflammations HANDICRAFTS: brooms	*Mushtekn*
*Boletus* spp. (Boletaceae)	*Këpurdha*	W	Fresh fruiting body	FOOD: stored dried and sold to middle men; traditionally it was not consumed, nowadays is sometimes used in omelettes with eggs and cheese, or as a filling for savory pies	
	(Varganj*)				
*Brassica oleracea* L. (Brassicaceae)	*Lakna*	C	Leaves	FOOD: in diverse preparations	*Lakna* FOOD: filling for savory pies; lactofermented, in *sarma* (sauerkraut leaves filled with rice and meat) or minced in salads
*Calamintha officinalis* Mill. (Lamiaceae)		W	Fresh leaves	MEDICINAL: externally applied to treat toothache	
*Cantharellus cibarius* Fr. (Cantharellaceae)	*Kepurdha*	W	Fruiting body	FOOD: consumed fried with eggs and clarified butter	
	(Lisiçarka*)				
*Capsicum annuum* L. (Solanaceae)	*Spec* (sweet varieties)	C	Dried fruits	FOOD: as a vegetable, fried; mixed with ricotta (*gjizë*) and consumed after a few weeks; ground, as one of the ingredients of the home-made seasoning mixture called *piprik e shtupun*, prepared by mixing ground red peppers, chilli, pumpkin seeds, corn flour, mint, and salt (traditionally consumed on boiled potatoes or warm bread)	*Spec*
	Piprik*	C	Dried fruits	FOOD: ingredient of the spice mix *piprik e shtupun* (see above)	
	(hot varieties)				
				MEDICINAL: ground and mixed with clarified butter or pork fat in a poultice, which is externally applied against rheumatisms	
				RITUAL: burned on the fire; the resulting strong odour is considered a repellent for werewolves (*lugata*)	
*Carlina acanthifolia* All. (Asteraceae)	*Thera*	W	Fresh flower receptacles	FOOD: consumed raw as snacks	
	Kaçani*				
*Carpinus betulus* L. (Betulaceae)	*Dru kaprivë*	W	Wood	HANDICRAFTS: diverse agricultural tools, including sickles	
*Carpinus orientalis* Mill. (Betulaceae)	Gaber*	W	Bark	VETERINARY: decoction, in external washed on cuts	
*Cetraria islandica* (L.) Ach. (Parmeliaceae)	*Mishk*	W	Thallus	MEDICINAL: gathered and traded in the past	
*Chenopodium bonus-henricus* L. (Amarathaceae)	Çuen*	W	Roots	FOOD: used in the past for making home-made halva* (Ottoman *sweet* prepared by gently stirring the decotion obtained by boiling these roots in water, with wheat and/or corn flour for one hour, and generally adding walnuts or raisins at the end, and letting it cool/solidify); the roots were also traded in the past	*Çuen* FOOD: home-made production of the sweet *halva,* made by cooking together roots, sugar syrup and powdered nuts - roots of *çuen* were erroneously identified by Doda as those of *Saponaria* spp. Upper Reka men were famous *halva*-sellers
*Citrullus lanatus* (Thunb.) Mansf. (Cucurbitaceae)	*Bostan*	B	Fruit pulp	FOOD/MEDICINAL: consumed raw, considered a means for cleansing the intestines	
	Lubenicë*				
*Clematis vitalba* L. (Ranunculaceae)	*Kurpna*	W	Branches	HANDICRAFTS: traditionally weaved in baskets used for bee-keeping	
	Pofit*				
			Fresh flowers	HONEY PLANT	
			(Dried?) flowers	FOOD: used in the past as bread yeast	
*Cornus mas* L. (Cornaceae)	*Thona*	W	Fresh fruits	FOOD: consumed raw; FOOD/MEDICINAL: syrups and distillate (*raki thonet*) considered healthy, esp. for treating fever	*Thon*
*Corylus avellana* L. (Betulaceae)	*Leithiza*	W	Kernels	FOOD: consumed raw as snacks	*Leithi*
			Branches	OTHERS: as structural supports for bean plants in the vegetable garden	
*Crataegus monogyna* Jacq. var. *sericea* Dzekov (Rosaceae)	*Murrisi*	W	Dried flowers	MEDICINAL: tea, as an anti-hypertensive	*Muris qeni* RITUAL: child affected by measles was placed under a hawthorn plant and water was thrown on him/her
			Fruits	FOOD: consumed as snack and in syrups and jams	
*Cucumis sativus* L. (Cucurbitaceae)	Kastraveca*	C	Fruits	FOOD: consumed raw, or, more often, lactofermented (turshi*)	
*Cucurbita maxima* Duchesne (Cucurbitaceae)	*Kungulla*	C	Fruits	FOOD: filling for pies	*Kungul* FOOD: filling for pies (*ndri*)
			Dried seeds	FOOD: consumed as snacks; ground and used as an ingredient of the home-made seasoning mixture *piprik e shtupun* (see *Capsicum annuum*)	
*Euphorbia* sp. (Euphorbiaceae)	*Lule gjarpi*	W	Aerial parts	OTHERS: crushed and used for fishing trout (*pastërmka*) in the river (as a fish poison)	*Lishanj*
*Fagus sylvatica* L. (Fagaceae)	*Ahu*	W	Fresh young leaves and kernels	FOOD: consumed as a snack in the past	*Ah*
			Branches and wood	FUEL	
				HANDICRAFTS: fences, diverse agricultural tools, “skeleton” of horse saddles and barns	
*Fomes fomentarius* (L.) J. J. Kickx (Polyporaceae)	*Eshka*	W	Dried fruiting body	OTHERS: burned; the resulting smoke is used to keep away bees while gathering honey	
*Fragaria vesca* L. (Rosaceae)	*Drezdha*	W	Fruits	FOOD: consumed raw	*Drethsa*
*Fraxinus excelsior* L. (Oleaceae)	*Frashëri*	W	Wood	HANDICRAFTS: for building flutes (kaval*)	
*Gentiana lutea* L. (Gentianaceae)	*Shtarë e egëra*	W	Roots	MEDICINAL: largely gathered and traded in the past; use unknown	*Shatra e egër*
*Helleborus* spp. (Ranunculaceae)	Kukurek*	W	Roots	MEDICINAL: inserted in the horse’s breast for treating muscular blocks (horses not able to be ridden anymore)	*Kukurek* VETERINARY: inserted into the nose to treat nasal congestion in horses
*Helichrysum plicatum* DC. (Asteraceae)	*Lule për molca*	W	Dried flowering tops	OTHERS: placed in the closets as a moth repellent	
*Hordeum vulgare* L. (Poaceae)	*Elb*	C	Fruits	FOOD: consumed in the past in gruels with corn; FODDER for sheep	*Elb*
*Hyosciamus niger* L. (Solanaceae)		W	Dried flowers	MEDICINAL: burned and the smoke exposed to the mouth to treat toothache (in the past)	
*Hypericum perforatum* L. (Hypericaceae)	Katrion*	W	Dried flowering tops	MEDICINAL: tea, for treating kidney stones, colds, stomach-ache, rheumatisms (used every day for at least a few months) or simply drunk as a “healthy” beverage; topically applied for treating wounds	
	Kantarion*				
	*Çaj bistrë*				
	*Lule e verdhë*				
			Fresh flowering tops	MEDICINAL: Macerate in oil (obtained by exposing it in the sun for several weeks) or prepare as a tea externally applied for treating skin burns, cuts, or other skin inflammations	
*Juglans regia* L. (Juglandaceae)	*Arra*	SD	Kernels	FOOD: used for cakes; a specific pie (*ndri*) was prepared with walnuts and lamb meat, and consumed on feast days	*Arr*
			Unripe fruits	FOOD/MEDICINAL: dipped in honey (and eventually lemon juice), the resulting preserve is considered healthy against tuberculosis and bronchitis	
*Juniperus communis* L. (Cupressaceae)	*Dëllinia*	W	Galbules	FOOD: seasoning MEDICINAL: tea, for treating cough, rheumatisms and “good for the blood”; largely gathered and sold, especially in the past	*Dulinj*
			Dried bark	OTHERS: smoked as a tobacco substitute	
*Lactuca sativa* L. (Asteracaeae)	Marolla*	C	Fresh leaves	FOOD: salads	
*Lycopersicon esculentum* Mill. (Solanaceae)	Patlixhan*	C	Fresh fruits	FOOD	*Patlingjan kuq*
*Malus domestica* Borkh. (Rosaceae)	*Molla*	SD	Fruits	FOOD/MEDICINAL: traditionally consumed raw, or roasted, or in pies or jams; the fruits of the most acidic landraces were used for producing home-made vinegar (adding water and letting ferment for 40 days) - this vinegar is considered healthy for treating hypertension	*Moll*
			Fruits→Raki	MEDICINAL: drunk as a stimulant (anti-lethargic)	
*Matricaria recutita* L*.* (Asteraceae)	*Kamomila*	W	Dried flowering aerial parts	MEDICINAL: tea for treating toothache, stomach-ache and belly pains (esp. in babies)	*Cfarlik*
*Medicago sativa* L. (Fabaceae)	*Jonxhe*	C	Aerial parts	FODDER	
*Melissa officinalis* L. (Lamiaceae)	*Milc*	W	Fresh flowers	HONEY PLANT: considered the best honey plant	
*Mentha longifolia* (L.) Huds. (Lamiaceae)	*Nagjas i egër*	W	Dried flowering tops	MEDICINAL: tea, as a stimulant (considered poisonous if drunk in large amounts)	
*Mentha spicata* L. (Lamiaceae)	*Nane*	W and C	Dried leaves	FOOD: ground, used as an ingredient of the seasoning mix *piprik e shtupun* (see *Capsicum annuum*)	
	*Nagjas*				
				MEDICINAL: tea, for treating stomach and intestinal pains, esp. in children, or as an anti-diarrhoeal	
*Nicotiana tabacum* L. (Solanaceae)	Duhan*	B	Dried crashed leaves	VETERINARY: externally applied on wounds or skin problems in sheep	MEDICINAL: external applications for treating wounds (mixed with honey)
	Tutun*				
*Orchis* spp. (Orchidaceae)	Salep* (two quoted “folk specifics”: one showing pink flowers and the other one with yellow flowers)	W	Dried tubers	MEDICINAL: ground, and then mixed with milk and dried again; the resulting powder is used in teas, as a “healthy” beverage (rarely macerated in plum distillate and drunk as a medicine); in the past largely gathered and sold	*Broçka Salep* FOOD: powdered orchid tubers were stirred with warm water and sugar; many young men from the upper Reka left their homes to work as *salep, bosa* and *halva* sellers in Skopje, Istanbul, Romania, and Bulgaria
*Origanum vulgare* L. (Lamiaceae)	Çaj*	W	Dried flowering aerial parts	MEDICINAL: tea for treating sore throat, cough, heart problems, intestinal discomforts, or as a recreational beverage	
	*Çaj i malit*				
	Çaj i livadhi*				
*Petasites hybridus* (L.) Gaertn. (Asteraceae)	*Kakuda Lapua*	W	Leaves	FODDER	*Kakuda*
*Phaseolus vulgaris* L. (Fabaceae)	Grosha*	C (brown and white landraces)	Dried seeds	FOOD: soups	*Grosh* FOOD: boiled, generally cooked together fresh or dried meat, adding bone marrow (*galgo*)
*Pisum sativum* L. (Fabaceae)	Grashaka*	C	Seeds	FOOD: cooked with meat or potatoes	*Nahut*
*Plantago major* L. (Plantaginaceae)	*Lule deli*	W	Leaves	MEDICINAL: tea, for treating kidney stones; externally applied for treating cuts	*Bajsht delit* MEDICINAL: external applications of leaves and roots for treating furuncles
*Primula veris* L. (Primulaceae)	Gornicfet*	W	Flowers	MEDICINAL: sold and traded in the past – use unknown	*Garicfet*
*Prunus avium* L. (Rosaceae)	*Shurshia*	SD	Fresh fruits	FOOD: consumed raw; syrups	*Qershi*
*Prunus cerasus* L. (Rosaceae)	Vishnja*	SD	Fruits	FOOD: consumed raw, or dried, or in syrups	*Vishnja*
			Resin (smol*)	MEDICINAL: externally applied on skin inflammations	
*Prunus cerasus* L. var. *marasca* (Host.) Viv. (Rosaceae)	*Shurshia e egër*	SD	Fruits	FOOD: consumed raw or dried, or in syrups	
*Prunus domestica* L. (Rosaceae)	*Kumbulla Gjagalka*	SD (many diverse landraces, with yellow, red, and black fruits)	Fruits	FOOD: consumed raw or dried; cooked with sugar and dried, and consumed as candies; hoshaf* – thickened fruit juice preserve; it is diluted with water (and eventually sugar) and drunk	*Kumla*
			Fresh fruits (fermented 1–2 months and then resulting must distilled)→raki*	MEDICINAL: instilled in the ear for treating earaches; drunk as a “healthy” beverage for the heart (rare) or to counteract tiredness; externally applied as a disinfectant for wounds	MEDICINAL: distillate externally applied on bullet wounds
*Pyrus communis* L. (Rosaceae)	*Dardha*	W	Fresh fruits	FOOD: consumed raw	*Dardha*
*Rhamnus alpina* L. (Rhamnaceae)		W	Fruits	FOOD: consumed as snacks	
*Robinia pseudoacacia* L. (Fabaceae)	Bagrem*	W	Fresh flowers	HONEY PLANT: the resulting honey is considered effective against cough	
*Rosa canina* L. (s.l.) (Rosaceae)	Kaça Shipinka*	W	Fresh fruits	FOOD: jams	*Kaç*
			Dried fruits	MEDICINAL: tea, for treating cold, fever, cough	
*Rubus idaeus* L. (Rosaceae)	*Medra*	W	Fresh fruits	FOOD/MEDICINAL: consumed raw; syrup (sok*) and hoshaf* (dense thickened juice, diluted with water and drunk) are considered healthy	*Medr*
	*Mjedra*				
	Malina*				
			Dried leaves	MEDICINAL: tea, for treating cold	
*Rubus schleicheri* Weihe ex Tratt. and other *Rubus* spp. (Rosaceae)	*Manaferra*	W	Fresh fruits	FOOD: consumed raw; jams	*Monca*
*Rumex acetosella* L. (Polygonaceae)	Gisilica*	W	Fresh and dried leaves	FOOD: filling for pies (in the past leaves were dried and stored for the winter, then rehydrated in water and used as a fresh vegetable)	*Gasilica*
	Kiselica*				
	Kisilica*				
*Rumex patientia* L. (Polygonaceae)	*Lepçeta*	W	Fresh leaves	FOOD: filling for pie (*peta*)	*Lipgjet* FOOD: consumed boiled with/in *dhalt* (kind of Albanian buttermilk)
*Salix alba* L. and other *Salix* spp. (Salicaceae)	*Shelçe*	W	Fresh branches	HANDICRAFTS: weaved in diverse kinds of baskets (kosh*)	*Shelçe* MEDICINAL: steam baths for treating rheumatisms
*Salvia verticillata* L. (Lamiaceae)	*Gamnash*	W	Fresh flowers	HONEY PLANT: The honey obtained from bees visiting the plant is considered very effective against bronchitis	
*Sambucus ebulus* L. (Adoxaceae)	Basdalina*	W	Fresh leaves	MEDICINAL: topically applied against snake bites	
	*Shtog i egër*				
*Sambucus nigra* L. (Adoxaceae)	*Shtog*	W	Flowers	FOOD/MEDICINAL: syrup (sok*) considered a cough remedy (expectorant); sometimes also given to children affected by belly pains to drink	*Shtog*
			Fresh fruits	FOOD: syrups and jams	
			Wood	HANDICRAFTS: for building spindles*	
*Satureja montana* L. (Lamiaceae)	*Lis*	W	Fresh flowers	HONEY PLANT	
*Secale cereale* L. (Poaceae)	*Thekna*	C	Fruits	FODDER	*Thekn* FOOD: *kurkurama* - gruel made by rye, corn, wheat and beans
				FOOD: roasted, as a coffee substitute*	
			Dried fruits (grounded)→Flour	FOOD: in the past used for baking sourdough bread (*bukë çerepi*) -prepared adding *dhallët* (buttermilk) and fermenting 2–3 days - and also for pies	FOOD: *buk thekninta –* sourdough bread; *buk e persiet –* sourdough bread made by mixing rye, wheat, and corn flours
			Dried aerial parts (straw)	HANDICRAFTS: filling for horse saddles, pillows and mattresses	*--*
*Sideritis* spp. (Lamiaceae)	*Çaj malit*	B (brought from the town *pazar*/market, presumably gathered from mountainous areas nearby)	Dried flowering aerial parts	MEDICINAL: tea for treating cold	
*Solanum tuberosum* L. (Solanaceae)	Repa*	C	Tubers	FOOD: traditionally consumed boiled with *piprik e shtupun* (see *Capsicum annuum*); fried, or roasted	*Kampire*
	Kompira*				
				MEDICINAL: slices of a fresh tuber were externally applied on the forehead for treating headaches	
			Young leaves	FOOD: boiled and consumed as vegetables with buttermilk, or as filling for pies (especially in the past – however one elderly couple confirmed that they also consume them nowadays)	
*Syringa vulgaris* L. (Oleaceae)	Ergovan*	C	Flowers	ORNAMENTAL	*Ergavan*
*Tanacetum vulgare* L. (Asteraceae)	Vratik*	W	Dried flowering tops	MEDICINAL: tea, as a digestive; in the past, the decoctions were externally used for washing children affected by rubella or persons affected by hepatitis* – for this last use sometimes the decoction was also drunk	
				VETERINARY: considered poisonous for calves	
				OTHERS: placed in closets as a moth repellent	
*Taraxacum officinale* Weber (Asteraceae)	*Bastë e egër*	W	Fresh leaves	FOOD: eaten in spring salads	
*Thymus serpyllum* L. (s.l.)	Lis Majçina dushnica*	W	Aerial parts	MEDICINAL: tea, for treating cold and cough	
(Lamiaceae)					
			Fresh flowers	HONEY PLANT	
*Tilia cordata* Mill. (Malvaceae)	Lipa*	SD	Dried inflorescences	MEDICINAL: tea, for treating colds	*Blini*
			Fresh flowers	HONEY PLANT	
			Resin (smol*)	MEDICINAL: externally applied to skin inflammations	
*Trifolium* spp. (Fabaceae)	Detelina*	W	Fresh flowers	HONEY PLANT;	*Trfonj*
				FODDER: for cows, it is considered a galactagogue (promoting milk production)	
*Trigonella foenum-graecum* L. (Fabaceae)	*Gruni piprikes*	C	Dried aerial parts	FOOD: as an ingredient of the seasoning mix *piprik e shtupun* (see *Capsicum annuum*)	
*Triticum aestivum* L. (Poaceae)	*Grur*	C	Fruits	FOOD	*Gruni* FOOD: *kukurama* - gruel made by rye, corn, wheat and beans
			Fruits (ground)→Flour	FOOD: bread and pies	FOOD: *buk e ngjeshun –* leavened bread; *buk grunit* – sourdough bread; *buk e persiet* – bread obtained mixing corn, rye, and wheat flours *peçiv -* kind of crusty bread, with a buttered inner part *fli* - a kind of crusty bread, made by several alternate layers of dough and butter, each layer is baked in sequence; *koleç* - bread made by diverse little bread units; *ndurdhi* - like *fli*, but with thicker layers, which are broken and finally dipped with melted butter *bosa* – a lacto-fermented beverage made with wheat flour, mixed with millet flour (or maize flour), which was boiled in water approx. 12 hrs.; the resulting mass was then knitted by hands and, after the adding of yeast, kept overnight, until it was dissolved in water; in the upper Reka, young men used to migrate to town as *bosa* producers and vendors in the Ottoman Empire
*Vaccinium myrtillus* L. (Ericaceae)	*Shurshia të egra*	W	Fresh fruits	FOOD/MEDICINAL: consumed raw, and sometimes believed to be “healthy for the blood”; syrups and jams; the fresh fruits are nowadays gathered in the summertime in large amounts and sold to middle men from Gostivar	*Qyrshiat t egra*
	*Baruk*				
	Borovnica*				
			Dried leaves	MEDICINAL: tea, used for heart problems	
*Veratrum album* L. (Melanthiaceae)	*Shtarë*	W	Roots	VETERINARY: decoctions, in external washes for treating lice in animals; root inserted in the horse’s breast for treating muscular blocks (horses can’t be ridden anymore)	*Shtar* VETERINARY: decoction of the roots was used for treating scabies in sheep
			Fresh leaves	VETERINARY: considered poisonous if animals consume them in large amounts (foaming at the mouth)	VETERINARY: Consuming large amounts of the leaves of the same plant was considered poisonous in sheep (foaming at the mouth), even very rarely lethal
			Dried leaves	OTHERS: smoked as tobacco substitute	
*Verbascum thapsus* L. (Scrophulariaceae)	*Bubujak Brusla*	W	Fresh leaves	MEDICINAL: externally as an haemostatic	*Bubujak*
				OTHERS: used for covering butter, peppers with ricotta cheese, or lacto-fermented vegetables	
*Urtica dioica* L. (Urticaceae)	Kapriva*	W	Fresh leaves	FOOD/MEDICINAL: consumed boiled (also in the past mixed with sorrel and potato leaves) or in soups, or as filling for savory pies – consumption of nettle is considered healthy as a “blood depurative” MEDICINAL: externally rubbed for treating rheumatisms	*Kopriva*
			Roots	FOOD: used in the past as rennet	
				MEDICINAL: decoctions are considered able to treat cancer and especially to relieve liver problems (decoction of the leaves and roots together)	
*Zea mays* L. (Poaceae)	*Çenk Kolomoç Barsak*	C (white and yellow landraces)	Fruits	FODDER	*Mçenk Kalamoç* FOOD: *kukurama -* gruel made by rye, corn, wheat and beans
			Dried fruits (ground)→Flour	FOOD: *buk kolomoçit* - bread (traditionally leavened with buttermilk [*dhallët*]); ingredient of the seasoning mix *bagrdar* - polenta obtained boiling the flour for at least one hour on the fire, generally served with buttermilk (*dhallët*), or clarified butter (*tlynë*) or yogurt (*kos*) - esp. ewe yogurt (*kos delje*); alternatively, polenta is served with beans or potato soup; pies (*peta*), filled with various vegetables	FOOD: *buk mçenkut* – bread; *buk pervlue* – sourdough bread; *pershenik*- leavened bread; *pershesh* - *pershenik* dipped in buttermilk [dhalt] or yogurt [kos]) *mçenka* (like *kukurama*, but prepared with corn only); *bagrdar* or *kaçamak me tlynë* - polenta served with clarified butter
				FODDER	
				RITUAL: corn flour was brought to the Islamic spiritual guide (*hoxha*), who “wrote” something with this; this was considered essential for treating the evil eye of a member of the family	
Various herbaceous species		W	Fresh stem	MEDICINAL: inserted into the anus, as a purgative	
Various tree species		W	Wood (burned) →Charcoal	MEDICINAL: used in the past in the ritual healing of the evil-eye: three pieces of hot coals were put in cold water; with the resulting water child face was washed (generally it has to be done by the first-born for his/her brothers/sisters; the first-born has to be treated by a neighbour) and the same water had to be drunk by the child or animal; depending on how the coal was dipped into water, this was also used for the diagnosis of the evil-eye – sometimes the water was given to the child in three spoons, which were then thrown behind the back; depending on how the spoons fell on the ground, the occurrence of the evil-eye was confirmed	
			Ash	OTHERS: for washing clothes	
Not identified	*Ferra magjara*	W	Leaves	FODDER: for donkeys	
Not identified	*Kulosgjarpni*	W	Fresh flowers	VETERINARY: applied externally against snake bites in horses	
Not identified	Morava*	W	Leaves	FOOD: filling for savory pies	

* Recorded local phytonyms, names of plant parts or plant preparations, which have been recorded also among South Slavs (even if the etymology may not be always Slavic; according to [22,34-44]); B: bought; C: cultivated; SD: semi-domesticated (not cultivated), but in some way “managed”; W: wild.

This seems to contradict what Bajazid Elmaz Doda postulated in his ethnographic report about the possible disappearance of the Albanians and their cultural heritage in the upper Reka [22], where an important folk medical heritage, although dramatically eroded, is still occurring. Among the most uncommon plant uses, the most noteworthy is the continuation of the use of the young leaves of cultivated potatoes and of wild *Rumex patientia* as filling for home-made savory pies. To the best of our knowledge, the recording of a food use of aerial parts of potatoes is new in contemporary Europe and may be explained by the extreme poverty and scarcity of resources in this mountainous area, even in the context of the Western Balkans. A confirmation of this phenomenon is perhaps best illustrated by the migration trends from the upper Reka to Romania and Istanbul (mainly of young men), beginning in the 19th Century [22]. In another study conducted on the Albanian side of Mount Korab (unpublished data), elderly locals confirmed that the upper Reka villages on the (current day) Macedonian side of the mountain were well known to them even in the folk history for being extremely disadvantaged in terms of socio-economic conditions.

### The linguistic features of the current ethnobotanical knowledge of the upper Reka Valley

In Table 1, the folk plant names that were recorded in the upper Reka Valley and which are also used by South Slavs are denoted by an asterisk. Approximately one-third of the recorded pythonyms are also used by the South Slavs, with some notable examples of Slavic etymology concerning culturally-important and very commonly used wild plants, such as *Urtica dioica, Hypericum perforatum*, and *Primula veris*, as well as most cultivated crops and some forest trees too.

### *Wild gentian* vs. *the white hellebore: a surprising cognitive “inversion”*

In the study area, the linguistic labels of gentian (*Gentiana lutea*) and white hellebore (*Veratrum album*) are the same. Gentian is, in fact, locally named as wild (meaning here “*looking-like”*) white hellebore (*shtarë*). This contradicts what would be expected regarding the plant cognitive prototype, which generally is represented by the most culturally salient or mostly used folk species [45], which in the Balkans is surely gentian. Instead, here gentian has been largely gathered solely for trade in the past and partially today, however a local use of gentian is unknown. Vice-versa, the use of hellebore in local ethnoveterinary practices may be very ancient; it was used mainly as external/topical agent for treating lice in diverse animals and especially for healing horses (roots were inserted into the musculature of the horse breast). This perhaps suggests that the gathering of *Veratrum album* in the Albanian mountains preceded the gathering of gentian, which could have been introduced by “external” factors: other cultures, such as the contiguous Slav ones, where the folk uses of gentian are widespread [1,4-7], or by the demands of urban markets.

### Cross-cultural comparison

**Figure 2:**
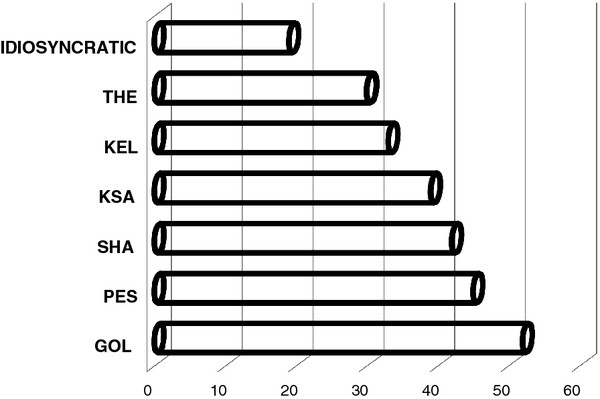
**Percentage of the wild medicinal plant taxa recorded and locally used in the upper Reka, which have also been recorded as used in field ethnobotanical studies conducted in other areas of Western Balkans (Figure**3**).**

**Figure 3:**
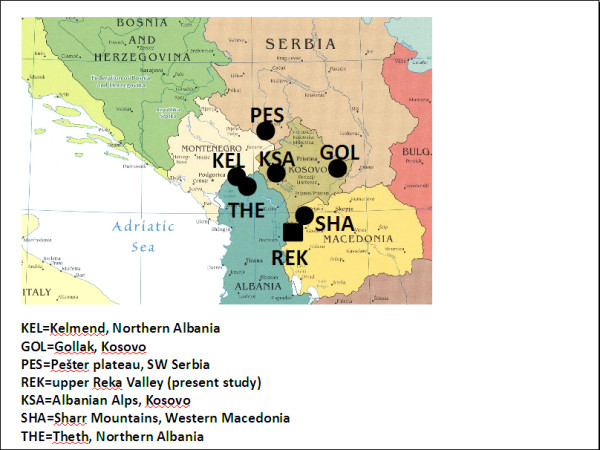
Location of the Western Balkan areas, where the ethnobotanical studies used for the comparative analysis have been recently conducted.

Figure 2 shows that a relevant portion of the medicinal plant taxa recorded and used in the upper Reka Valley are also part of the folk medical heritage of surrounding Balkan regions, where other field ethnobotanical surveys have been recently conducted (Figure 3).

This is especially true in those areas where the Albanian population was historically in extensive contact with the South-Slavic cultures, such as the Gollak area in eastern Kosovo [9], the Pešter plateau in south-western Serbia [1] and the Sharr Mountain (*Šar Planina* in Macedonian) in western Macedonia [29] (Figure 3).

This may confirm the findings of both our linguistic analysis on the folk plant names carried out in Table 1 and also Franz Nopcsa’s ethnolinguistic analysis of the terms referring to the material culture in upper Reka [22], which showed very important loans from the Romanian and especially Slavic languages. It can thus be postulated that the upper Reka Albanians had been heavily influenced by the Slavic culture - and not vice-versa, as Spiridon Gopčević stated [23].

Study participants confirmed that over recent decades their most important markets and “exchange” centres have been the multi-ethnic (Macedonian, Albanian, and Turk) towns of Gostivar in Western Macedonia and Prizren, in Southern Kosovo. Moreover, it must also be noted that over the span of the last century, the Albanians of the upper Reka lived outside of the borders of the Albanian state (founded in 1912), and for the major part of this period within the former Socialist Republic of Macedonia within Yugoslavia, where the dominant culture and languages have been Macedonian and Serbo-Croatian. In other words, the remarkable “interference” of the Slavic cultures found within the domain of Albanian traditional plant knowledge of the upper Reka represents a unique phenomenon, which nowadays is not easy to trace back in detail. This could be due to the difficulty faced in establishing to which degree the Slavic culture influenced the traditional knowledge among Albanians in the upper Reka, considering the role that ancient “hybridisations” may have played, as both Gopčević and Nopcsa, although in a different way, have underlined in their respective works.

Moreover, as well analysed by Fredrick Barth more than four decades ago [46], cultural contacts and boundaries among ethnic groups may be very complex and subject to dynamic change, since they respond to very unique societal and historical circumstances. It could be interesting to follow the future development of local perceptions of nature among the last remaining Albanians of the upper Reka and the strategies that they will adopt through processes of further negotiation of their identities within the rest of the population in Western Macedonia and the whole country.

### Other domestic remedies

Table 2 reports other domestic and medicinal remedies recorded in the area, which are not based on indigenous plants; a large portion of these remedies survives only in the memories of the interviewees.

**Table 2:** Food, medicinal, and other domestic uses of non-indigenous plants, and animal, mineral, and industrial products quoted in the study area

***Product (local name)***	***Local use***
Animal rennet (stomach of very young animals) (*sirisht*)	Used for producing cheese, but also as a starter for making yogurt#; anti-diarrheal
Ants	Used in the past as a rennet substitute#
Bear’s fat	Used externally for treating rheumatisms#
Beer	One glass of beer, drunk, is considered healthy for the kidney
Black piece of cloth	Tied onto cow’s neck or horns, as a protective amulet against evil eye#
Bullet	Attached to clothes and worn as a protective amulet against evil eye#
Buttermilk (*dhallët*)	Drunk as a post-partum reconstituent or for treating intestinal troubles and hypertension; used as starter for producing home-made yogurt
Chicken	Cooked for a long time, until obtaining a gelatinous material, which is further cooked together with onions, corn flour and vinegar to create home-made soap#
Clarified butter (*tlynë*)	Drunk for treating hypotension
Clothes dressed on the wrong side	Protective amulet against evil eye#
Coffee powder	Spoonful is ingested for treating hypotension; decoction (“Turkish coffee”) for hypotension; externally applied to cuts
Copper sulphate	Used externally for healing lameness in sheep#
Cow’s milk	Drunk in cases of constipation
Cut	Cutting the ewe’s ear and letting blood coming out was considered an effective method for treating several sheep diseases#
Dried sheep and cow’s faeces	Burned, the resulting smoke keeps the bees away while taking honey#
Goat milk	Applied (warm) into the ear against earache#
Gunpowder (*barut*)	Its odour is exposed to the nose of sleepwalkers, in order to bring them back to consciousness#; odour was also considered a repellent for werewolves#
Hare’s meat	If consumed, believed to inhibit fertility#
Honey (*mjalt*)	Consumed for improving blood circulation or as a post-partum reconstituent: Ingested for treating sore throats
Knife	A knife placed under the pillow is considered preventive for sleepwalking#
Leech	Applied externally for “sucking the bad blood”#
Lemon	Drunk to treat hypertension; sometimes used in the past as rennet for making cheese#
Match’s head	Topically applied for treating toothaches#
Mother’s milk	Instilled in the ear for treating inflammations/earache
Mud	Applied onto bee stings for pain relief#
Oil	Ingested to treat constipation
Pork fat	Externally used on burns#
Propolis	Tea or macerate in *raki* used for treating cough/respiratory problems and intestinal discomforts (all of which are considered “new” uses)
Ricotta cheese (*gjizë*)	Consumed, is considered “good for the blood”
Royal gelly	Consumed for improving mental faculties (“new” use)
Salt	Brought to the Islamic spiritual guide (*hoxha*), who “wrote something” with this# - this was considered essential for treating the evil eye of a member of the family; mixed with water, and the resulting solution instilled in the ear or eye for treating inflammations; mixed with hot water in external bathes for treating chilblains;
	Applied topically for treating toothache
Soap	A small piece inserted in the anus, as a purgative#
Snow	Applied on the feet for relieving arthritic pains
Starch	Ingested for treating diarrhoea
Stone	Pressed on skin zone affected by the bee bite, in order to relieve the pain
Sugar	Externally applied to cuts; mixed with water (*sherbet*) for treating stomach-ache; burned and ingested considered a medicine for sore throats
Tobacco	Haemostatic
Urin (human urine)	Externally applied on cuts#; drunk against hepatitis#
Vinegar from honey (*uthull dëgjetes*) - produced at home fermenting in water honey and raw wax for a couple of weeks	Used as rennet#; Externally applied on the front or feet for treating fever; applied on the chest for treating bronchitis; applied on the belly of babies when crying or colicky
Yogurt (*kos*)	Post-partum reconstituent
Water	Drunk against high blood pressure; Fumigations of hot water (eventually heated by previously heated stone) for treating cold
Whey (*hirra*)	Drunk as a diuretic, or against hypertension, or “to decrease fats in the blood”
Wool	Raw sheep wool externally applied for treating bruises#

# remembered, but nowadays disappeared use(s).

## Conclusions

The very few last remaining Albanians living on the Macedonian side of Mount Korab of the upper Reka still retain a remarkable level of local knowledge concerning botanicals; this knowledge is however eroded, especially in quantitative terms, due the very tiny population, who have decided to remain in the region despite the influence of economic hardships. The hybrid “Albanian-Slav” cultural features of the local inhabitants, which have been largely discussed and disputed in Balkanological studies, could be confirmed in our ethnobotanical surveys, since both local plant names and especially a significant portion of the recorded plant uses share common features with other Slavic and culturally mixed areas of the Western Balkans. The multi-faceted knowledge recorded here could represent a crucial added value for the local managers of the Mavrovo National Park and also for further fostering new forms of eco-tourism, which must be sensitive not only to local biodiversity, but also to the multi-cultural dimension of a historically complex area like the upper Reka.

## Competing interests

The authors declare that they have no competing interests.

## Authors’ contributions

AP designed the research and conducted the historical and field studies; BR assisted in the field study; AN, VK, and HA contributed to the ethnolinguistic and cross-cultural comparative analysis of the data; AN, HA, BM, and KC analysed the botanical taxonomic part of the data; AP and CLQ drafted the overall scientific discussion. All authors read and approved the final manuscript.
